# Transobturator midurethral sling: What should patients expect after surgery?

**DOI:** 10.1007/s00192-017-3408-2

**Published:** 2017-07-08

**Authors:** Tomasz Rechberger, Andrzej Wrobel, Alicja Zietek, Ewa Rechberger, Michal Bogusiewicz, Pawel Miotla

**Affiliations:** 2nd Department of Gynaecology, Medical University of Lublin, ul. Jaczewskiego 8, 20-954 Lublin, Poland

**Keywords:** Stress urinary incontinence, SUI, Mixed urinary incontinence, MUI, Lower urinary tract symptoms, LUTS

## Abstract

**Introduction and hypothesis:**

Midurethral sling (MUS) surgeries are minimally invasive procedures; however, they are not free of postoperative complications. The aim of the study was to assess the occurrence of lower urinary tract symptoms (LUTS) (urgency, nocturia, frequency, splitting/spraying, hesitancy, terminal dribbling, and subjective feeling of postvoid residual) in patients suffering from stress (SUI) or mixed (MUI) urinary incontinence with a predominant SUI component before and after transobturator MUS placement.

**Methods:**

The study group consisted of 88 women with SUI and 18 with MUI who underwent transobturator MUS. All participants were questioned with a self-developed questionnaire before and after surgery regarding the presence of LUTS.

**Results:**

Seven days after surgery, 62 patients (58.5%) noted voiding and postmicturition symptoms, whereas 67 (63.2%) reported problems in storage. The more commonly reported LUTS at week 1 after surgery were urgency (52.8%), splitting/spraying (41.5%), and feeling of incomplete bladder emptying (34.0%). Patients perceived that splitting/spraying was the most bothersome. After 6 months, the most common LUTS reported were hesitancy (14.1%), terminal dribbling (10.4%), and splitting/spraying (9.4%). We noticed a decrease in the number of urgency episodes >2.7 times (*p* < 0.001) compared with baseline. After 6 months, 97 (91.5%) patients reported the lack of incontinence episodes.

**Conclusions:**

A vast majority of patients after MUS suffer from LUTS in the early postoperative period; however, the majority of undesired symptoms resolve spontaneously within the first 6 months postsurgery.

## Introduction

Since its introduction into clinical practice in 1995, midurethral sling (MUS) has become a common anti-incontinence procedure for stress urinary incontinence (SUI) worldwide (currently, an estimated rate of 9.45 cases per 10,000 women) [1]. Indeed, the changing female demographic trend has been accompanied by a significant increase in the number of SUI sufferers, most of whom report experiencing negative consequences on their quality of life (QoL), and some epidemiologists predict even a > 50% increased demand for mitigating urogynaecological services over the next 40 years [2, 3]. Due to its technical simplicity, MUS is widely performed by physicians of different specialities, mainly, urologists and gynecologists, with some country-specific differences [4, 5].

Although all types (retropubic, transobturator or single-incisions) of slings are considered as being minimally invasive procedures with a relatively low morbidity rate, they are not free of either intraoperative or postoperative complications [6]. Nevertheless, a considerable number of patients report various postoperative lower urinary tract symptoms (LUTS), which have a significant impact on the final evaluation of the procedure’s effectiveness [7]. Recently, a published detailed analysis of 8772 patients from the National Prospective Database who had undergone MUS procedures focused on the 30-day morbidity and reoperation rate. This was stratified additionally by the specialty of performing surgeon (urologists and gynecologists) [8]. The authors found that the overall 30-day rate of any complications was as low as 3.52%. Taking these data into consideration, it is obvious that any unwanted LUTS occurring after anti-incontinence surgery will decrease patients’ satisfaction, and variable information concerning LUTS occurrence and its natural evolution after the MUS procedure is of pivotal clinical importance. The aim of our study was to estimate the presence and de novo occurrence of LUTS before and after transobturator MUS placement in patients suffering from stress (SUI) or mixed (MUI) urinary incontinence with a predominant SUI component, and its natural evolution (first 7 days, 6 weeks, and 6 months after surgery). We also investigated patients’ postoperative satisfaction with surgery using the Likert scale [9].

## Materials and methods

The study protocol was approved by our local institutional ethical committee (KE-0254/74/2015). All participating patients gave written informed consent. Women were eligible for the study if they had predominant symptoms of SUI as revealed by a positive cough test either in supine or standing positions at bladder volume ∼250–300 ml, bladder capacity ≥250 ml, and postvoid residual (PVR) ≤ 50 ml, without clinically relevant pelvic organ prolapse [Pelvic Organ Prolapse Quantification system (POP-Q) ≤ 1]. Study exclusion criteria were evidence of obstructed voiding in the absence of prolapse, severe comorbidities, and previous pelvic surgery. Based on these criteria, the study was conducted from November 2015 to May 2016 on 106 women, all of whom had undergone surgical treatment for SUI (*n* = 88; 83%) or MUI with a predominant SUI component (*n* = 18; 17%). The objective severity of SUI symptoms were assessed using the Sandvik scale before and after surgery during initial and follow–up visits [10]. All participants were questioned with a self-developed questionnaire (Appendix) before and after surgery to determine the presence, persistence, or new occurrence (de novo) of common LUTS. This was divided into storage (urgency, increased daytime frequency, nocturia), voiding (slow stream, splitting/spraying, intermittency, hesitancy, straining, terminal dribbling), and postmicturition (feeling of incomplete emptying) symptoms according to the classification proposed by Abrams et al. [11]. LUTS were ascertained by interview, with symptoms rated positive if they occurred more than three times a week. All patients had undergone an ambulatory transobturator MUS procedure (T-sling, Herniamesh, Italy) with additional two-point tape fixation. Absorbable fixating sutures were placed parallel to the urethra 0.5 cm laterally on each side of the midurethra and between 1 and 1.5 cm from the external urethral meatus to prevent tape displacement during the healing period [12]. Before discharge, all patients were assessed using abdominal and introital ultrasonography (postvoid residual and tape position, respectively) and free-flow uroflowmetry to exclude the possibility of bladder outlet obstruction (BOO). Follow-up visits were scheduled 7 days, 6 weeks, and 6 months after surgical intervention. The Likert scale of seven grades was used to evaluate the level of patients’ satisfaction after treatment [9]. This psychometric scale is commonly involved in research that employs questionnaires, and participants specified their level of agreement or disagreement on a symmetric agree/disagree scale regarding satisfaction.

In our study, statistical analyses were performed with Statistica package version 12.0 (StatSoft Inc., Tulsa, OK, USA). A *p* value <0.05 was considered statistically significant. The chi-square test was used as statistical test and was applied to all sets of categorical data to evaluate how likely it is that any observed difference between the sets arose by chance.

## Results

**Fig. 1:**
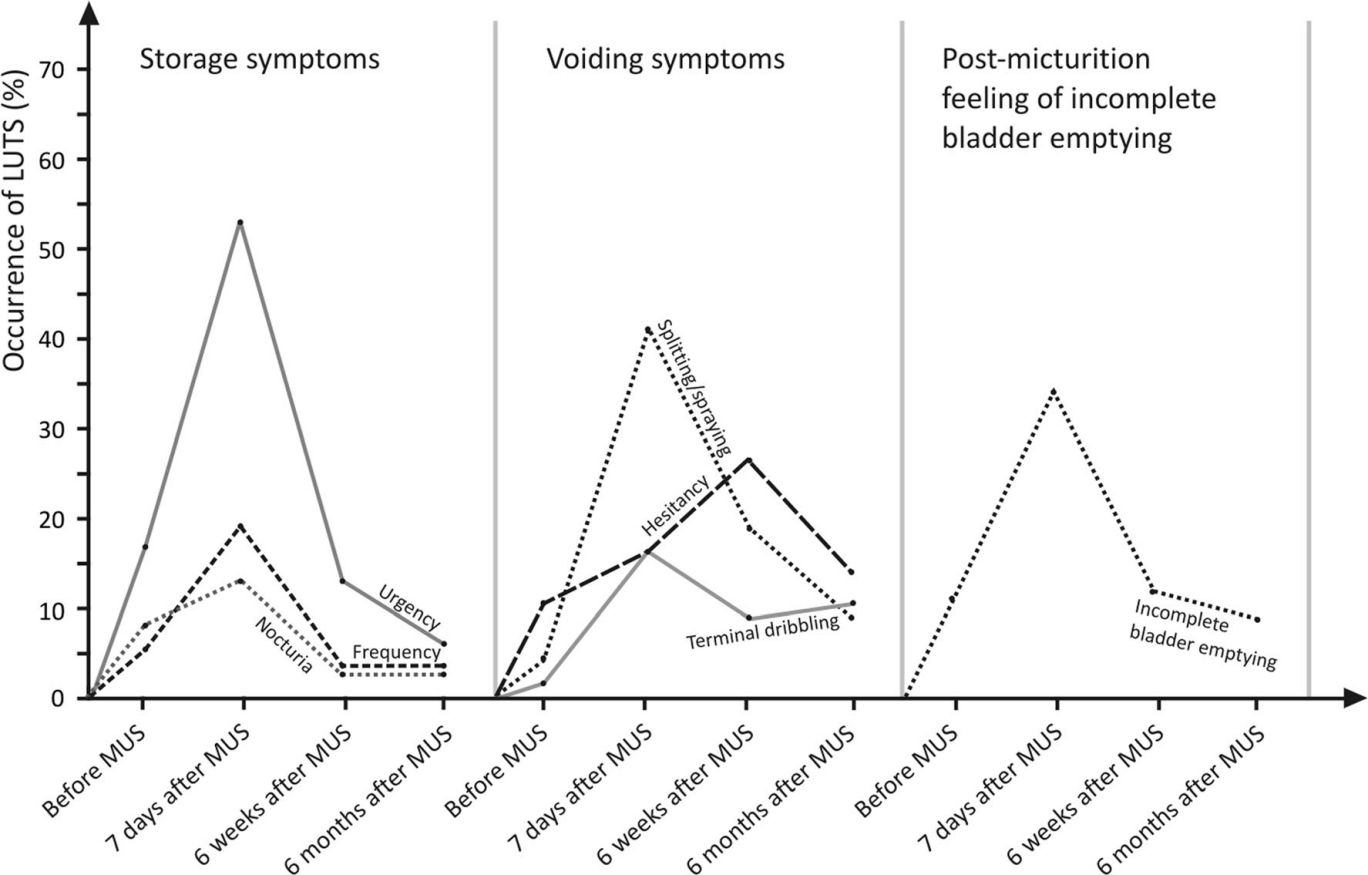
Natural course of lower urinary tract symptoms (LUTS) after midurethral sling surgery (MUS)

Mean patient age was 49.1 ± 10.6 years; 56 were premenopausal and 50 postmenopausal. The average parity was 1.9 ± 0.8, and average body mass index (BMI) was 27.2 ± 3.4. All participants were discharged home within 4–6 h after surgery after first spontaneous voiding (PVR < 50 ml). The only intraoperative complication was vaginal epithelial perforation, which was repaired during the primary procedure (*n* = 3). Based on the Sandvik scale, 80 (75.5%) patients before surgery had severe and 26 (24.5%) moderate incontinence. After 6 months, 97 (91.5%) patients reported the lack of incontinence episodes, eight (7.5%) still had moderately severe SUI, and one had no improvement at all. All patients with moderate severity had severe incontinence prior to MUS surgery. Seven days after surgery, 62 patients (58.5%) reported voiding and postmicturition symptoms, and 67 (63.2%) made mention of storage problems [18 (17%) patients had both]. The most commonly reported LUTS at week 1 after surgery were urgency (52.8%; threefold increase from baseline), splitting/spraying (41.5%; almost ninefold increase), and postvoid feeling of incomplete bladder emptying (34%; threefold increase). Nocturia was the least frequent symptom (13.2%). Surprisingly, patients considered splitting/spraying as being the most bothersome, and although the incidence decreased to 9.4% at month 6, it remained twice as high in comparison with baseline. Detailed data concerning LUTS occurrence and evolution in the postoperative period are summarized in Table 1 and Fig. 1. The mean values of maximum flow and voided volume were 24.7 ml/s (±10.5) and 314 ml (±74.4), respectively; we observed no correlation between postoperative uroflowmetry and occurrence of storage symptoms.

**Table 1:** Lower urinary tract symptoms (LUTS) before midurethral sling surgery (MUS) and at follow-up (FU) visits

Symptoms		Before MUS* n* (%)	7 days after MUS* n* (%)	6 weeks after MUS* n* (%)	6 months after MUS* n* (%)	Differences between follow-up periods		
						Before vs. 7 days FU (*p* value)	Before vs.6 months FU (*p* value)	7 days vs. 6 months FU(*p* value)
Storage symptoms	Urgency	18 (17.0)	56 (52.8)	14 (13.2)	7 (6.6)	<0.001	=0.02	<0.001
	Frequency	6 (5.7)	20 (18.9)	4 (3.8)	4 (3.8)	<0.001	NS	<0.001
	Nocturia	9 (8.5)	14 (13.2)	3 (2.8)	3 (2.8)	NS	NS	=0.006
Voiding symptoms	Splitting/ spraying	5 (4.7)	44 (41.5)	20 (18.9)	10 (9.4)	<0.001	NS	<0.001
	Hesitancy	11 (10.4)	18 (17.0)	29 (27.4)	15 (14.1)	NS	NS	NS
	Terminal dribbling	2 (1.9)	18 (17.0)	15 (14.1)	11 (10.4)	<0.001	=0.01	NS
Postmicturition feeling of incomplete bladder emptying		12 (11.3)	36 (34.0)	13 (12.2)	9 (8.5)	<0.001	NS	<0.001

At the 6-month follow-up, patients were asked to evaluate the level of satisfaction according to the Likert’s scale of seven grades. Seventy-four patients (69.8%) indicated 7, which means they were fully satisfied. Two (1.9%) declared little improvement and rated their satisfaction as 3. The most common LUTS after 6 months were hesitancy (14.1%), terminal dribbling (10.4%), and splitting/spraying (9.4%). Interestingly, the incidence of all these symptoms was higher than preoperatively. We had, however, also observed that patients reported a decrease of urgency >2.7 times when compared with baseline visit.

Among participants with MUI, of 18 patients who suffered from urgency symptoms before treatment, 16 still reported urgency 7 days after surgery, three at 6 weeks’ follow-up, and one after 6 months. In the six patients reporting frequency before the procedure, this symptom remained 7 days after MUS. Only two patients from this group complained of frequency in the 6-week and 6-month follow-up. In two patients with terminal dribbling prior to MUS, one complained of its presence in the follow-up observation period. In the 12 patients with feelings of incomplete bladder emptying, ten claimed it persisted at 7 days after MUS, which decreased to three after the 6-week and 6-month follow-up. Among 11 patients with hesitancy before the surgery, this symptom remained at the 7-day follow-up, but only one woman reported it at the 6-week follow-up; this symptom was not reported at the 6-month follow-up. Splitting/spraying was present in five patients before and 7 days after MUS; however, the symptom disappeared completely by the end of the observation period. None of the nine patients with nocturia noted this symptom after MUS.

## Discussion

To the best of our knowledge, this is the first study to assess prospectively the natural resolution of predefined LUTS after MUS. Due to their reproducible, good long-term outcomes and their relatively low morbidity rate, MUS operations have become the most commonly performed and most studied surgical procedures for SUI over the past decade. It is known, however, that general patient satisfaction after MUS surgery can be affected by many factors, including inherent patient-specific expectations, postoperative continence status, and unwanted complications related to surgery. The methodology of clinical evaluation after sling surgeries remains controversial. The pathophysiology of these symptoms seems to be connected with an increase in urethral resistance and irritation of the urethra caused by the tape [13]. The prevalence of subjective LUTS varies in different populations, with urinary urgency being the most prevalent, as it was in our study group [14]. However, various LUTS after incontinence surgery are well-described complications common to all stress incontinence procedures. Nevertheless, various LUTS are often not followed up for an adequate duration in sling trials, and it has been challenging to accurately estimate their rates due to loss to follow-up [15]. Moreover, in some published reports, there is no information regarding the presence of preoperative storage or voiding symptoms, both of which may act as confounding factors when evaluating postoperative complication rates and patient satisfaction after surgery. Still, it is known that LUTS after suburethral sling surgeries can be the major factor in patient dissatisfaction and can even lead to subsequent complications. Symptoms reported by patients involve issues in the storage phase (increased frequency, urinary urgency, nocturia, and urgency incontinence), voiding (hesitancy, straining, weak stream, terminal dribbling) and in postmicturition (urinary retention). Patients might also suffer from bladder pain, dysuria, or urinary tract infections (UTI). In the case of total urinary retention, the detrusor muscle cannot overcome the urethral resistance induced by the sling. On the other hand, when postoperative patients experience no total retention, but reported frequency, urgency, or urgency incontinence with or without poor urinary flow, it is difficult to establish an accurate diagnosis [16]. Urinary retention is usually diagnosed when the patient requires catheterisation within 28 days after the surgery [17]. It seems that the increase in urethral resistance is an indisputable risk factor of LUTS. Moreover, it is likely that the sling might evoke detrusor overactivity that manifests in the case of increased urine volume [18].

Previous reports have described the predictive values of baseline demographic and clinical factors and urodynamic measures on surgical efficacy or voiding dysfunction among patients who participated in SISTEr trials, which compared outcomes between Burch colposuspension and pubovaginal autologous fascial sling. Detailed analysis on preoperative subjective voiding symptoms in our participants revealed that the most commonly was bending, and the most common abnormality of urinary stream was dribbling after urination. However, only hesitating stream was a predictor of both postoperative LUTS, overall failure, and stress-specific failure [19]. In addition, some urinary stream characteristics (spurting and abnormal streams) were significantly associated with stress-specific failures, whereas symptoms of physical accommodation to facilitate voiding (strain, bend, lean, stand, press, push, or other) did not predict postoperative dysfunction or surgical failure.

In a recently published analysis of 30-day morbidity and reoperation rates following MUS, the most common reason for readmission was urinary symptoms; however, few patients were subsequently diagnosed with UTI. This situation indicates that voiding dysfunctions and not infection was the consequence of the surgery itself [8]. In fact, in our study, no patient was hospitalized again during the 6-month follow-up. We also found no postoperative UTI during the follow-up period, which we believe was the result of antibiotic treatment for 5 days postsurgery that accompanied any case of clinically relevant urinary retention.

In several reports, incomplete bladder emptying at discharge after MUS varied from 10% to 24%, whereas in our material, all patients at discharge had PVR < 50 ml, despite some patients having had the feeling of incomplete bladder emptying [7, 20]. According to previously published reports, preoperative urodynamic parameters did not influence the risk of incomplete bladder emptying, whereas symptoms such as the need to stand to urinate, to press on the bladder, or to push on the vagina more than doubled the risk [20]. Still, the severity of incontinence at baseline—both objective (as measured by incontinence episode frequency, pad test weight) and subjective (as measured by the Incontinence Impact Questionnaire and Urogenital Distress Inventory score)—was not statistically different between satisfied and unsatisfied patients after MUS, despite the fact that significant improvement of other symptoms, such as urgency and frequency, were reported by most participants as well [21]. This was also the case in our study group. Others observed that the main significant risk factor for the development of LUTS after MUS surgery was a preoperative peak urinary flow rate < 20 ml/s [7], as well as an increased preoperative value of postvoid residual, with no difference in voiding trial failures between retropubic and transobturator groups [22].

In a long-term observational study, the authors assessed LUTS and risk factors for an unsatisfactory outcome after retropubic slings. Two years after surgery, 3.6% of patients reported the development of de novo urgency, which increased to 10.8% at 5 years and to 14.4% at 10 years. Therefore, the authors concluded that overactive bladder (OAB) symptoms are the main factor in unsatisfactory long-term outcomes in MUS surgeries [23]. In another multicenter prospective study, at 10 years follow-up, 14% of women with pure SUI treated by transobturator MUS also reported symptoms of de novo OAB. Such results underline the notion that MUS surgery itself is an independent risk factor for subsequent OAB development [24].

We believe that our study adds to the existing literature on the influence of MUS on pre-existing LUTS among SUI patients. Besides providing relevant data, the study contributes important information on general patient satisfaction in relation to pre-existing or de novo LUTS after surgery. Accordingly, by assessing patient impression of severity and perceived improvement as assessed by Sandvik and Likert scales, we found that patients’ global impression of improvement was highly predictive of being “mostly” or “completely” satisfied. The strength of this study is that data were collected prospectively with a predefined list of symptoms to monitor; however, definite limitations are its single-site setting, limited number of patients, and lack of standardized validated questionnaires.

In the first 6 weeks after MUS, >60% of women will experience some undesired LUTS. As these are probably inherently connected with this type of surgical intervention, patients should be informed that such undesired symptoms could occur in the first few weeks after intervention but will probably undergo natural resolution. Since the most frequent LUTS after MUS was urgency, it will be of great interest whether simple prophylaxis with antimuscarinics or βeta-3- mimetics in the early postoperative period will decrease the percentage of women suffering from such an unwanted symptom.

## Appendix

**Lower urinary tract symptoms questionnaire**

(Translated from the original Polish)

Please fill out the following seven questions to your best ability.

All the terms will be explained.

If you have any questions, please let us know.

Thank you.

Questions on the occurrence of lower urinary tract symptoms (LUTS):
